# What do ultrasound vocalizations really mean in rats with different origins of pain?

**DOI:** 10.1097/PR9.0000000000001230

**Published:** 2024-12-24

**Authors:** Yang Yu, Chun-Li Li, Rui Du, Xiao-Liang Wang, Jun Chen

**Affiliations:** a Institute for Biomedical Sciences of Pain, Tangdu Hospital, The Fourth Military Medical University, Xi'an, P. R. China; b Sanhang Institute for Brain Science and Technology, Northwestern Polytechnical University, Xi'an, P. R. China

**Keywords:** Ultrasound vocalization, Pain models, Lidocaine, Amitriptyline, Gabapentin, Rat

## Abstract

**Objectives:**

This study is to assess how 22 kHz and 50 kHz spontaneous ultrasound vocalization (USV) calls would be affected by different origins of pain so as to validate the use of USV in pain studies.

**Methods:**

Five well-established rat models of pain were used to evaluate various parameters of spontaneous 22 kHz and 50 kHz calls in adult male rats in terms of both acute and chronic or inflammatory and neuropathic or somatic and visceral origins. The effects of local lidocaine blockade of the injection site and intraperitoneal administration of antidepressant (amitriptyline) and anticonvulsant (gabapentin) were examined as well in typical inflammatory and neuropathic pain models, respectively.

**Results:**

The major new gains were as follows: (1) naive rats staying alone and engaging dyadic social interaction with a naive or a conspecific in pain emitted similar power and amounts of both 22 kHz and 50 kHz spontaneous USV calls; however, rats suffering from various origins of pain emitted significantly less USV calls of both 22 kHz and 50 kHz in terms of both number and time. (2) Local blockade of the injury sites of inflammatory pain could reverse the impaired emission of both 22 kHz and 50 kHz spontaneous calls, so did the treatment of the rats with neuropathic pain by amitriptyline and gabapentin.

**Conclusions:**

Emissions of both 22 kHz and 50 kHz spontaneous calls were impaired by acute and chronic pain conditions regardless of inflammatory and neuropathic or somatic and visceral origins.

## Abstract 2

Supplemental Digital Content is Available in the Text.

Emissions of spontaneous ultrasonic vocalizations are impaired by both acute and chronic pain regardless of inflammatory and neuropathic or somatic and visceral origins.

## 1. Introduction

In clinic, patients with both acute and chronic pain lose their daily activities, social communications, and working abilities because of negative mood, bad sleep, and low quality of life. Social communication in mammals, including humans, is mainly based on innate vocal sounds (eg, laughing, crying, or screaming) that may be affected by pain. Acoustic signals are the oldest interindividual signaling system in vertebrates.^8^ In recent years, a certain pattern of frequency spectrum of ultrasound vocalization (USV), a measure of emotional state and social communications of rodents, has been increasingly used for the evaluation of pain. Adult rats emit 2 types of USV calls in emotionally distinct contexts. The first type is 22 kHz vocalization, with frequencies across 18 to 32 kHz, serving as an indicator of negative emotional states.^14,33^ Two subtypes of 22 kHz USV calls have been identified: (1) long 22 kHz calls (≥300 ms) serving as alarming signals of external or potential danger; (2) short 22 kHz calls (<300 ms) expressing a state of discomfort or distress without external source of danger.^10^ The second type is 50 kHz vocalization, with frequencies across 35 to 70 kHz, serving as an indicator of positive, appetitive, or hedonic affective state and social communication.^14^ Fourteen sonographic patterns of 50 kHz vocalizations have been identified, and 3 sonographic patterns (flat, trills, and step-trills) have been mostly studied.^63^

In pain research, measurement of pain is largely based on evoked responses to an external acute stimulation, eg, a series of von Frey filaments rating the paw withdrawal mechanical threshold and radiant heat stimuli rating the paw withdrawal thermal latency. However, pain is a multidimensional experience involving sensory discrimination, emotional motivation, and cognitive evaluation; paw withdrawal responses cannot fully reflect animals' pain experience, especially the emotional or spontaneous nature of pain in rodents.^12,32^ Therefore, it is essential to develop new quantitative analysis of spontaneous behaviors associated with pain, adding new biomarkers representing inner pain state and processing.^32^ Ultrasound vocalization has been considered as a mature measurement of the emotional response in rats.^30,31^ Some previous studies have shown that pain affects the emission of 22 kHz and/or 50 kHz USV calls in rats with spontaneous pain^1,3–6,12,22,28,32,34,37,40–44,47,53^ or evoked pain.^2,15,17,18,20,21,23–27,48–50,52,54,55,57–62^ However, conclusive recommendations have yet been given because of discrepancies among the previous results even using the same animal model of pain (for details see supplementary Table S1, http://links.lww.com/PR9/A276). For example, using the formalin test, some reports demonstrated increase in the number of 22 kHz calls,^6,29,53^ whereas other reports showed no change.^52,56,62^ These controversial results were also true for 50 kHz calls using the same orofacial formalin test.^3,6^ The reasons for these discrepancies are largely unknown and may be due to the use of limited parameters, patterns, time course, and methods for USV analysis. Moreover, selection of pain models and assays may also matter in terms of acute or chronic, inflammatory or neuropathic, somatic or visceral, and spontaneous or evoked pain.^38,51^

To validate the use and appropriately interpret the meanings of the USV calls in pain studies, in this study, 5 well-established rat models of pain were used to evaluate various parameters of spontaneous 22 kHz and 50 kHz calls in adult male rats with naive staying alone or engaging dyadic social interaction served as controls. Identification of USV calls in rats submitting to different pain models may represent a new approach for understanding the brain circuitry underlying rodents' affective states and a useful tool in the development of appropriate pharmacological therapies.^6^ Surprisingly, it was interesting to find that emissions of both 22 kHz and 50 kHz spontaneous USV calls were all impaired by both acute and chronic persistent pain conditions regardless of inflammatory and neuropathic or somatic and visceral origins.

## 2. Methods

### 2.1. Animals

The experiments were conducted in male and female Sprague-Dawley albino rats (6 weeks old, 150–180 g) bought from the Laboratory Animal Center of the Fourth Military Medical University (FMMU, Ref No.: IACUC-20200955). Animals were cohoused in a group of 4 to 6 rats in each cage at the specific-pathogen-free (SPF) animal facility, which was automatically controlled under a light/dark cycle (08:00–20:00/20:00–08:00) with a humidity of 40 to 60% and a room temperature of 22 to 26°C for 2 weeks after arrival at Tangdu Hospital.^64^ The food and water were taken by the animals ad libitum. Animals were treated in accordance with the recommendations of the ARRIVE guidelines^36^ and the U.K. Animals (Scientific Procedures) Act 1986 and associated guidelines, the EU Directive 2010/63/EU for animal experiments, the National Institutes of Health guide for the care and use of laboratory animals (NIH Publications No. 8023, revised 1978), and the ethical guidelines for investigations of experimental pain in conscious animals of the International Association for the Study of Pain were also critically followed.^66^ All experiments were conducted between 9 am and 12 am, and animals were randomly assigned to the different groups.

### 2.2. Experimental design

All rats were randomly divided into 10 groups: (1) single naive control (Single_Naive_): rats staying alone without any treatment (n = 8 for male and n = 6 for female); (2) dyadic social interaction between a naive and a naive conspecific (dyadic social interaction [DSI]_Naive-Naive_, n = 6 pairs for both male and female); (3) dyadic social interaction between a naive and a conspecific in bee venom (BV)–induced pain (DSI_Naive-Pain_, n = 6 pairs for male only); (4) BV model: rats received subcutaneous (s.c.) injection of BV (0.2 mg in 50 μL, Sigma, St. Louis, MO) into the left hind paw (n = 8 for male only); (5) formalin (F) test: rats received s.c. injection of formalin solution (2.5%, 100 µL) into the left hind paw (n = 8 for male only); (6) acetic acid (AA) model: rats received intraperitoneal (i.p.) injection of 0.9% AA (10 mL/kg body weight, n = 8 for male only); (7-8) complete Freund adjuvant (CFA) model: rats received s.c. injection of CFA solution into the left hind paw (100 µL, 1:1 dissolved in physiological saline, Sigma) and were divided into 2 groups (n = 8 for each male group only); (9-10) spared nerve injury (SNI) model, rats received surgical transection of both the tibial and peroneal nerves but with the sural nerve intact and were divided into 2 groups (n = 8 for each male group only). All rats received 30-minute USV recordings in a sound-proof chamber; those with BV, F, and AA were arranged to receive USV recordings immediately after treatment, whereas those with CFA or SNI received USV recordings 0.5 hours (CFA-0.5h) and 24 hours (CFA-24h) or 14 days (SNI-14d) and 30 days (SNI-30d) after preparations. For preparation of the animal models used here see previous literature.^16,38,39,51,64^

### 2.3. Drugs

Anticonvulsant gabapentin (GBP) (50 mg/kg, i.p., n = 8 for male only)^19^ or antidepressant amitriptyline (AMI) (50 mg/kg, i.p., n = 8 for male only)^35^ was administered in rats with neuropathic pain, whereas lidocaine (0.2 mg in 50 μL, n = 8 for male only, Shanghai Harvest Pharmaceutical Co., Ltd, China) was administered in rats with inflammatory pain. Physiological saline was used as vehicle for the above drugs.

### 2.4. Apparatus and ultrasound vocalization recordings

Spectrum of USV was recorded using the MED USV Detector (NL-937-1, Med Associates Inc) with a manufacturer's default settings with 2 preset bandwidths of 18 to 32 kHz and 35 to 70 kHz, a threshold of 35 dB and a minimal gap of 0.06 seconds between USVs. The MED USV Detector was positioned in a sound-proof chamber (100 cm × 40 cm × 30 cm) in which a rat cage (40 cm × 15.5 cm × 24 cm) was included. Sonograms were visualized using the MED USV Viewer through which real-time sequential USV data containing the number, time, and duration (offset minus onset of an ultrasonic signal, in ms) were exported as text file for off-line and statistical analyses. The original peak frequency (Hz) of an individual USV call, namely, its strongest frequency component that had the highest amplitude,^7,65^ was also calculated as required. Specifically, 50 kHz USV was defined as calls with frequencies across 35 to 70 kHz, whereas 22 kHz USV was defined as calls with frequencies across 18 to 32 kHz. Specifically, 22 kHz USVs were calls with duration <300 ms (frequency < 32 kHz), whereas calls with duration ≥300 ms (frequency < 32 kHz) were referred to as long 22 USV calls.^11^

In this study, only spontaneous USVs were studied, and the following parameters were adopted^7^: (1) time sequential changes in power (in dB), the intensity of each USV calling over 30-minute time course; (2) time changes in call rate, 30-minute time course of the number of calls per 5 minutes, which represents the time-related magnitude of calling; (3) total number of USVs within a period of 30 minutes; (4) total time of USVs within a period of 30 minutes; (5) duration of an ultrasonic signal (in ms); and (6) bandwidth of USVs, including 22 kHz calls and 50 kHz calls.

### 2.5. Statistical analysis

All data were expressed as means ± SEM. Statistical analysis was performed using GraphPad Prism version 8.0 (GraphPad Software Inc., CA) and IBM SPSS Statistics version 25.0 software (IBM Inc). One-way ANOVA with LSD post hoc corrections and independent sample *t* test (2-tailed) were used for parametric data, whereas Kruskal–Wallis 1-way ANOVA test with Bonferroni post hoc corrections and Mann–Whitney *U* test were used for non-parametric data according to the results of normality Shapiro–Wilk test and equal variance Levene test (for details see Supplementary Table S2, http://links.lww.com/PR9/A276). *P* < 0.05 was considered statistically significant.

## 3. Results

### 3.1. Assessment of 22 kHz and 50 kHz spontaneous calls in rats under different pain conditions

#### 3.1.1. Changes in powers of 22 kHz and 50 kHz spontaneous calls in rats with different origins of pain

**Figure 1.**
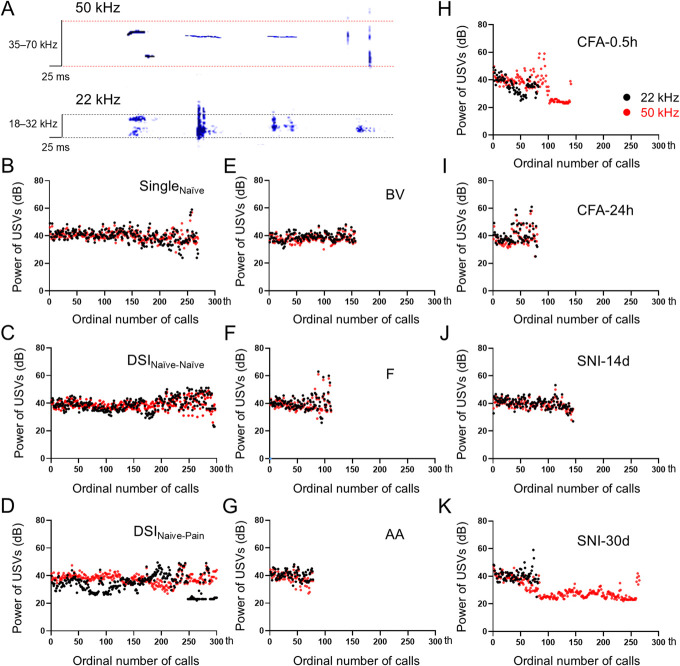
Ultrasonographic signal examples of and the effects of social stimulation and pain on powers of spontaneous USVs calls (dB) in adult male rats. (A) Examples of ultrasonographic signals of 22 kHz and 50 kHz calls. (B-D) changes in powers of 22 kHz and 50 kHz calls during 30-minute recordings from rats staying alone (Single_Naive_) and those engaging dyadic social interaction (DSI) with a naive (DSI_Naive-Naive_) or a conspecific in pain (DSI_Naive-Pain_). (E-K) Changes in powers of 22 kHz and 50 kHz calls during 30-minute recordings from rats after receiving subcutaneous injection of bee venom (BV), formalin (F), acetic acid (AA), and complete Freund adjuvant (CFA) or spared nerve injury (SNI) preparation. One dot represents 1 ultrasonographic signal seen in (A); n = 8 animals for each single group and n = 6 pairs of rats for DSI. CFA-0.5h and CFA-24h, 0.5 hours and 24 hours after CFA injection; SNI-14d and SNI-30d, 14 and 30 days after SNI preparation; USV, ultrasound vocalization.

**Figure 2.**
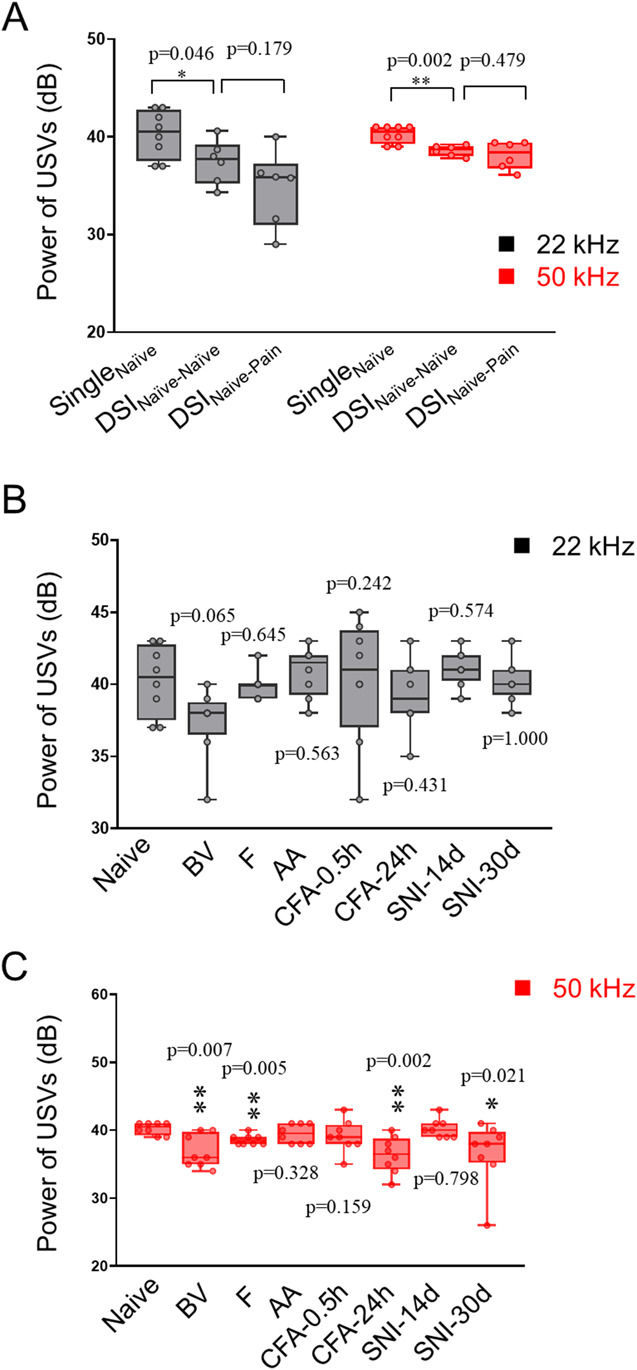
Averaged powers of spontaneous USV calls in single naive, DSI, and pain-suffering rats. (A) Comparisons of USV powers between Single_Naive_ and DSI_Naive-Naive_ and DSI_Naive-Pain_ groups. (B, C) Comparisons of USV powers between single naive rats and rats after receiving subcutaneous injection of bee venom (BV), formalin (F), acetic acid (AA), and complete Freund adjuvant (CFA) or spared nerve injury (SNI) preparation. n = 8 animals for each single group and n = 6 pairs of rats for DSI. *P < 0.05, **P < 0.01 vs single naive group. CFA-0.5h and CFA-24h, 0.5 hours and 24 hours after CFA injection; DSI, dyadic social interaction; SNI-14d and SNI-30d, 14 and 30 days after SNI preparation; USV, ultrasound vocalization.

Figure 1A showed examples of the major subtypes of ultrasonic signals of both 22 kHz and 50 kHz in adult male rats. There was no significant difference in power, total number, and time of either 22 kHz or 50 kHz spontaneous calls between male and female rats under single naive (Single_Naive_) and DSI_Naive-Naive_ paradigms (supplementary Figure S1, http://links.lww.com/PR9/A276). Thus, we conducted the following experiments only in male rats. Naive male rats staying alone (Single_Naive_) emitted similar amounts of 22 kHz and 50 kHz spontaneous calls to those of DSI_Naive-Naive_ and DSI_Naive-Pain_ groups (Figs. 1B–D). However, male rats with various pain conditions emitted significantly less spontaneous calls of either 22 kHz or 50 kHz relative to the single naive control (Figs. 1E-J). However, as for SNI-30d, the emission of 50 kHz calls remained similar to naive control, although the emission of 22 kHz calls was impaired (Fig. 1K). Statistical analyses showed significant reduction in powers of 22 kHz and 50 kHz calls in DSI paradigms relative to single naive control (Fig. 2A). The powers of 50 kHz calls were only reduced in BV, F, CFA-24h, and SNI-30d groups relative to single naive control, although pain effect on powers of 22 kHz calls was not seen (Figs. 2B and C and supplementary Table S2, http://links.lww.com/PR9/A276).

#### 3.1.2. Changes in number, time, and sonographic duration of 22 kHz and 50 kHz spontaneous calls in male rats with different origins of pain

**Figure 3.**
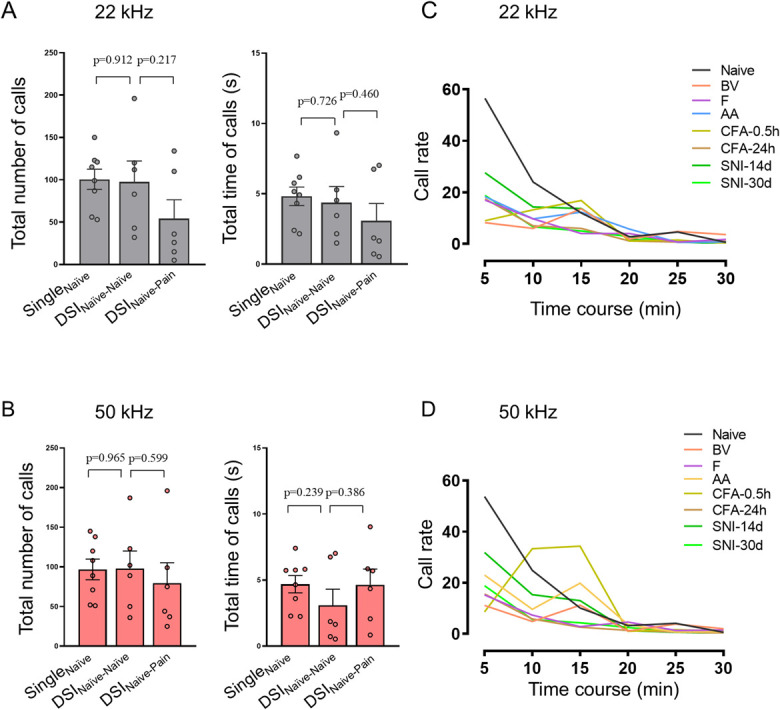
Comparative analysis of USV calls between single naive and DSI groups and the time course effects of pain on emission of USV calls. (A-B) Comparisons of averaged total number and time of 22 kHz and 50 kHz spontaneous calls between Single_Naive_, DSI_Naive-Naive_, and DSI_Naive-Pain_ groups. (C-D) Time courses of 22 kHz and 50 kHz spontaneous call rate emitted from single naive rats and those received different pain treatments. Data expressed as mean ± SEM; n = 8 animals in each single group and n = 6 pairs of rats for DSI. CFA-0.5h and CFA-24h, 0.5 hours and 24 hours after CFA injection; DSI, dyadic social interaction; SNI-14d and SNI-30d, 14 and 30 days after SNI preparation; USV, ultrasound vocalization.

**Figure 4.**
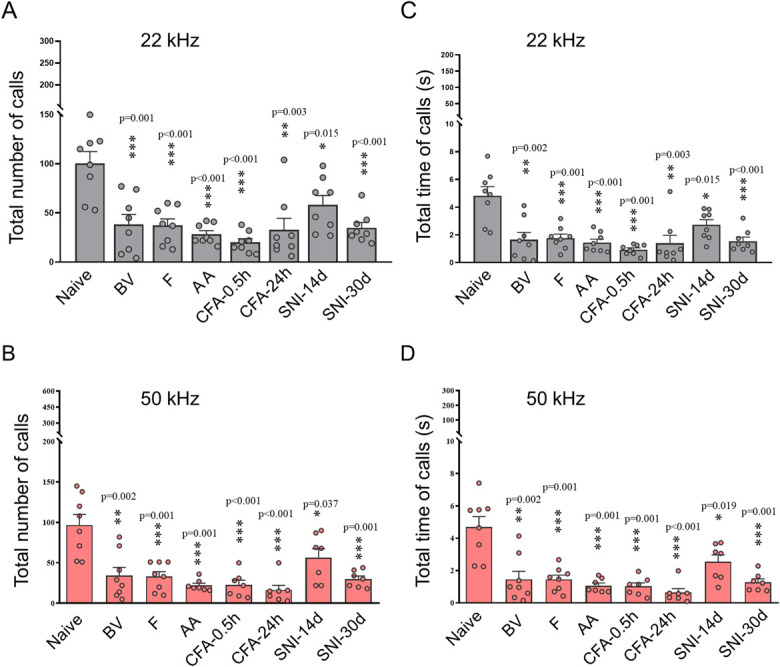
Comparative analysis of USV calls between single naive rats and those received different pain treatments. (A-D) Comparisons of the total number and time of 22 kHz and 50 kHz calls between single naive rats and those after receiving injection of BV, F, AA, and CFA or SNI preparation. Data expressed as mean ± SEM; n = 8 animals in each group; *P < 0.05, **P < 0.01, ***P < 0.001 vs Naive rats. AA, acetic acid; BV, bee venom; CFA, complete Freund's adjuvant; CFA-0.5h and CFA-24h, 0.5 hours and 24 hours after CFA injection; SNI-14d and SNI-30d, 14 and 30 days after SNI preparation; USV, ultrasound vocalization.

Generally, among 88 male rats (including DSI groups) tested, 94.32% emitted only short 22 kHz and 50 kHz USV calls regardless of what pain model or test used. Only 5.68% (5 of 88) of rats emitted long 22-kHz calls among which only 1 rat was encountered from each of the 5 groups (AA, CFA-0.5h, CFA-24h, SNI-14d, and SNI-30d), respectively. There were no rats emitting long 22 kHz calls in Single_Naive_, DSI_Naive-Naive_, DSI_Naive-Pain_, BV, and F groups. There was no significant difference in the total number and time of 22 kHz and 50 kHz spontaneous calls between Single_Naive_ and DSI_Naive-Naive_ and DSI_Naive-Pain_ (Figs. 3A and B). Time course analysis showed distinct decrease in call rate of both 22 kHz and 50 kHz spontaneous calls in rats with various origins of pain relative to single naive control (Figs. 3C and D). The overall USV calls in naive rats were most abundant during the first 5 minutes and diminished gradually over the remaining 25-minute period. Compared with the naive control, the changes in call rate for both 22 kHz and 50 kHz calls were more significant in the first 5-minute time block. However, for CFA-0.5h group, the call rate for 50 kHz, but not for 22 kHz, was very low in the first 5-minute time block but reached the highest level during 10 to 15 minutes (Fig. 3D). Statistical analyses further showed significant differences in the total number and time of both 22 kHz and 50 kHz USV calls between single naive and rats with different origins of pain (Fig. 4 and Table S2, http://links.lww.com/PR9/A276). The sonographic duration per USV call was not sensitive to most groups except BV, CFA-24h, and SNI-30d in which the USV call durations were significantly shortened relative to single naive control (Fig. S2 and Table S2, http://links.lww.com/PR9/A276).

### 3.2. Reversal effects of antinociceptive, antidepressant, and anticonvulsant drugs on impaired ultrasound vocalization calls in male rats with inflammatory and neuropathic pain

**Figure 5.**
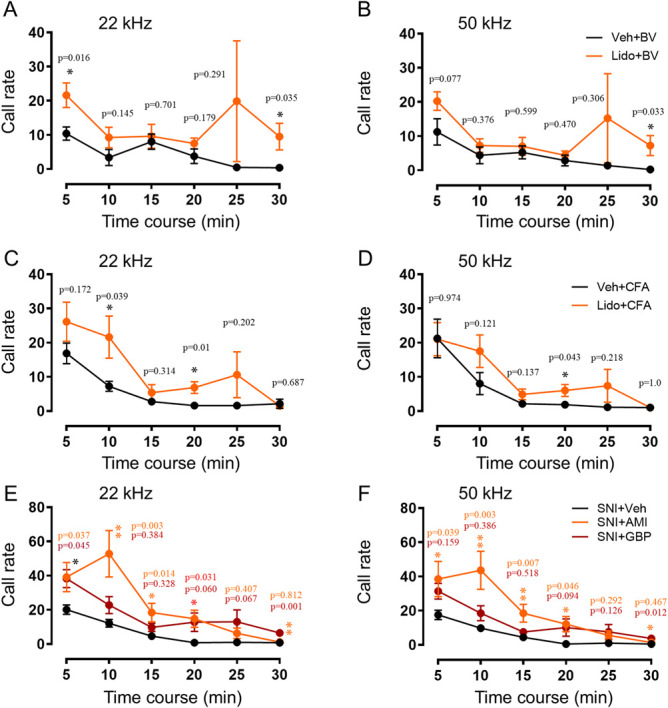
Effects of antinociceptive, antidepressant, and anticonvulsant drugs on impaired USV calls in rats with inflammatory and neuropathic pain. (A-D) Time course effects of local lidocaine (Lido) blockade of BV or CFA injection sites on impaired emission of 22 kHz and 50 kHz spontaneous calls by inflammatory pain. (D, E) Time course effects of intraperitoneal administration of amitriptyline (AMI) and gabapentin (GBP) on impaired emission of 22 kHz and 50 kHz calls by SNI-induced neuropathic pain. Data expressed as mean ± SEM; n = 8 animals per group; *P < 0.05, **P < 0.01, vs Veh. BV, bee venom; CFA, complete Freund adjuvant; USV, ultrasound vocalization.

**Figure 6.**
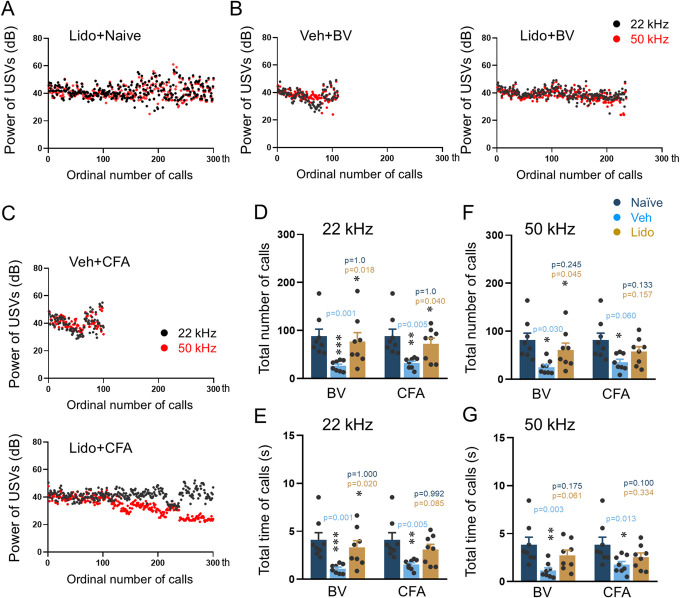
Reversal effects of local lidocaine (Lido) blockade of peripheral inflammatory pain on impaired emission of spontaneous USVs calls in rats. (A-C) Reversal effects of local Lido blockade of BV and CFA injection sites on impaired emission (powers) of 22 kHz and 50 kHz spontaneous calls caused by peripheral inflammatory pain. (D-G) Reversal effects of Lido blockade of BV and CFA injection sites on impaired emission (number and time) of 22 kHz and 50 kHz calls caused by peripheral inflammatory pain. Data expressed as mean ± SEM; n = 8 animals per group; *P < 0.05, **P < 0.01, ***P < 0.001 vs naive or Veh. BV, bee venom; CFA, complete Freund adjuvant; USV, ultrasound vocalization.

**Figure 7.**
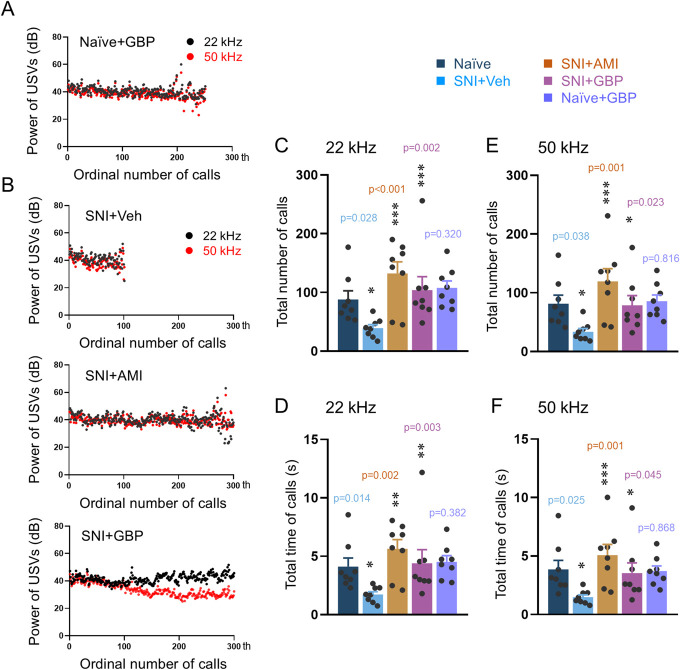
Reversal effects of antidepressant and anticonvulsant treatment of neuropathic pain on impaired emission of spontaneous USVs calls in rats. (A, B) Reversal effects of amitriptyline (AMI) and gabapentin (GBP) on impaired emission (powers) of 22 kHz and 50 kHz spontaneous calls caused by SNI-induced neuropathic pain. (C-F) Reversal effects of AMI and GBP on impaired emission (number and time) of 22 kHz and 50 kHz calls caused by SNI-induced neuropathic pain. Data expressed as mean ± SEM; n = 8 animals per group; *P < 0.05, **P < 0.01, ***P < 0.001 vs naive or Veh. SNI, spared nerve injury; USV, ultrasound vocalization.

Local lidocaine administration at both BV and CFA injection sites and systemic administration of AMI and GBP in male rats with SNI-induced neuropathic pain could reverse the impaired 22 kHz and 50 kHz USV calls, respectively, relative to vehicle controls (Fig. 5). Lidocaine treatment of naive male rats did not change either the power or the time sequence of 22 kHz and 50 kHz USV calls (Fig. 6A). Preblockade of the BV and CFA injection sites with lidocaine resulted in full restoration of the impaired emission of USV calls to the normal level in terms of total number, time, and sonographic duration of both 22 kHz and 50 kHz calls (Figs. 6B–G, Fig. S3A,B and Table S2, http://links.lww.com/PR9/A276). Systemic treatment with GBP and AMI (not shown) did not influence the emission of 22 kHz and 50 kHz spontaneous calls in single naive rats (Fig. 7A). Therapeutically, both AMI and GBP resulted in full reversal of the impaired emission of both 22 kHz and 50 kHz calls relative to vehicle control (Figs. 7B–F, Fig. S3C,D and Table S2, http://links.lww.com/PR9/A276).

## 4. Discussion

### 4.1. What do ultrasound vocalizations really mean in rats with different origins of pain?

In this study, we gained in several aspects for further understanding about the meaning and interpretation of various parameters of USV calls associated with pain using 5 well-established models representing different etiologies of pain in terms of both acute and chronic or inflammatory and neuropathic or somatic and visceral origins. Actually, evaluation of multiple parameters of spontaneous USVs using different pain models made a difference in understanding about how pain influences the emission of 22 kHz and 50 kHz spontaneous calls in rats. First of all, we compared 30-minute emissions of spontaneous USV calls between single naive (Single_Naive_) and 2 DSI paradigms (DSI_Naive-Naive_ and DSI_Naive-Pain_) and found that (1) rats of both single naive and DSI paradigms could emit 22 kHz (short only) and 50 kHz spontaneous calls in similar patterns; (2) there was no significant difference in number and time of either 22 kHz or 50 kHz spontaneous calls between single naive and DSI paradigms; (3) there was no sex-related difference in number and time of either 22 kHz or 50 kHz spontaneous calls under both Single_Naive_ and DSI_Naive-Naive_ paradigms; (4) the powers of both 22 kHz and 50 kHz were weakened by the 2 DSI paradigms relative to Single_Naive_ in male rats, whereas female rats showed increased power of 22 kHz, but not 50 kHz, under DSI_Naive-Naive_ than Single_Naive_; (5) social transferring of pain could not be detected by USV measurement because no significant difference was seen between DSI_Naive-Naive_ and DSI_Naive-Pain_ in terms of power, number, and time of spontaneous USV calls, although social buffering effect on pain could not be excluded due to the inability of the USV detector to discriminate individual emission of ultrasonic signals from DSI. Taking these results together, it is suggested that social stimulation does not play a determining role in induction of either 22 kHz or 50 kHz spontaneous USV calls, although the powers of these spontaneous USV calls can be changed by the social interaction. Thus, it is inappropriate to define short 22 kHz USV calls as an aversive or dangerous call as what has been done for long 22 kHz calls before.^9,10,13,14,33,63^ Burgdorf et al.^14^ have proposed a circadian cycle of affect in rats by USV responses, including sonographic pattern of the calls to different social stimulation (hedonic or aversive tickling), namely, from neutral (Flat 50 kHz) to hedonic appetitive (Trill 50 kHz) and to hedonic consummatory (Step 50 kHz) and to aversive avoidance (20 kHz) and finally to sleep. In the radio model of affect, hedonic tickling during wake cycle time causes hedonic appetitive and consummatory 50 kHz calls (from Trill to Step sonographic patterns) with inverse “U” shape, whereas, however, aversive tickling (forced arousal and activity during sleep) causes aversive 22 kHz calls.^14^ Because the subtypes (short or long) of 22 kHz calls have not been clarified in most of the previous studies, including those studies for pain (also see supplementary Table S1, http://links.lww.com/PR9/A276), it is not clear whether the so-called aversive 22 kHz calls are long or short or both. In this study, nonetheless, we clarified that long 22 kHz USV calls were not seen in either single naive or the 2 DSI paradigms during 30-minute recordings. Furthermore, the emissions of short 22 kHz and 50 kHz USV calls in single naive and DSI rats are not likely to be alarming or aversive in nature because emissions of both short 22 kHz and 50 kHz USV calls are greatly decreased or impaired in rats in pain of different origins. Actually, in more recent studies, decrease in the number of 22 kHz and 50 kHz calls has been reported in orofacial formalin, CCI, and LPS-induced cerebral inflammatory pain models,^3–5,12,47^ supporting what we found here. Regarding the pain-related decrease in USV callings, Brudzynski has proposed that a vocalizing rat subjected to acute pain ceases to make ultrasonic calls, likely because of the magnitude of anxiety but not the physical nature of the aversive stimulus.^7^ This may somewhat explains our and some previous experimental findings.

Moreover, there have existed some reports failing to record any changes in spontaneous 22 kHz and/or 50 kHz calls in formalin-, carrageenan-, CFA-, and streptozotocin-induced diabetic neuropathic pain models.^6,32,52,56,62^ The reasons for the failure in capturing changes in spontaneous UVS calls are not clear; nonetheless, missing out on parameters, short-time course recording, and poor designs may lead to undetermined and unconvincing results. Conversely, increased number of 22 kHz and/or 50 kHz calls have also been reported in various animal models of pain,^6,15,22,34,37,40–44,53,61^ and the reasons for the discrepancies between those previous and our current results are not clear either and possibly due to differences in experimental design, use or no use of external stimuli, parameters, types, and time of USV recordings, which are also required to be further studied. Innocuous and noxious external stimuli in rats with both arthritic and neuropathic pain have been demonstrated to be able to evoke increases in both audible (20 Hz-16 kHz) and USV (25 ± 4 kHz) vocalizations (see supplementary Table S1, http://links.lww.com/PR9/A276),^2,17,20,21,23–27,48–50,54,55,57–60^ suggesting that external stimuli to rats with pain of both inflammatory and neuropathic origins may evoke emissions of both audible and long 22 kHz calls; however, these may not be expressed in rats when external stimuli are not supplied even when they are under some pain conditions. This phenomenon is like the situation in patients with pain. When suffering from pain caused by injury, the patients would decrease their daily activities, oral communications, and body languages due to unpleasant sensory and emotional experiences; however, if the injury site were stimulated by some external stimuli they would vocalize, scream, or cry due to intolerable hyperalgesia or allodynia.

Here, we did not examine sex effects and failed to analyze sonographic patterns of 22 kHz and 50 kHz calls in rats with different origins of pain. However, these limitations would not negate the conclusions made here regardless of whether the sonographic patterns of USVs are changed or not.

### 4.2. Can ultrasound vocalization measures be used in evaluation of preclinical pain in rodents?

One of the most interesting points of studying USVs in rodents is to use it in evaluation of preclinical pain for R&D of new pain-killing drugs. However, so far, the use of USVs for this purpose is yet determined due to discrepancies in results across different previous studies. To see whether the changed powers and decreased number and time in 22 kHz (short) and 50 kHz calls seen here can be useful in R&D of new pain-killing drugs, the sensitivity of these changes to conventional analgesic treatments was examined pharmacologically. In practice, lidocaine blockade of the injection sites of BV and CFA, 2 well-established peripheral inflammatory pain models, was conducted before BV or CFA injections. The purpose of the use of peripheral blockade by lidocaine but not by systemic opioids or nonsteroidal anti-inflammatory drugs was to simply avoid central effects of these drugs so as to make clear whether the USV changes seen in this study are caused by peripheral nociceptive input but not by pain-related emotional comorbidities (anxiety or depression).^45,46^ Here, peripheral preblockade of nociceptive input from the injection site by lidocaine was able to prevent the decrease in emissions of both 22 kHz (short) and 50 kHz USV calls from occurring, suggesting that persistent nociceptive input from the periphery may impair the functions of the neural circuits for the emission of 22 kHz (short) and 50 kHz calls. Moreover, we also examined the effects of systemic administration of AMI, an antidepressant, and GBP, an anticonvulsant, both of which are first-line clinical drugs proofed by FDA for the treatment of neuropathic pain, in rats with neuropathic pain. The results showed that treatment of rats with SNI-induced neuropathic pain condition by AMI and GBP could also reverse the decreased emission of both 22 kHz (short) and 50 kHz USV calls to the normal level, suggesting that central inhibition of painful information arising from peripheral nerve injury by AMI and GBP may restore the impaired functions of the neural circuits for the emission of 22 kHz (short) and 50 kHz USV calls as well.^19^ It has been well established that the neural activity of the ascending mesolimbic cholinergic system, which originates from a neuronal subpopulation of the laterodorsal tegmental nucleus, generates aversive 22 kHz calls, whereas that of the ascending mesolimbic dopaminergic system, which originates from a neuronal subpopulation of the ventral tegmental area, generates appetitive (hedonic) 50 kHz calls.^14^ So far, the interactions between the ascending pain systems and the 2 ascending mesolimbic systems for emissions of 22 kHz and 50 kHz calls under different pain conditions are not clear and worthy of being further studied in details. Based on our current pharmacological data, it is suggested that pain-related impairment of emissions of spontaneous 22 kHz (short) and 50 kHz calls can be used as a secondary end point for preclinical evaluation of new drugs for the treatment of different origins of pain in rats.

## Disclosures

The authors have no conflict of interest to declare.

## Appendix A. Supplemental digital content

Supplemental digital content associated with this article can be found online at http://links.lww.com/PR9/A276.

## Supplementary Material

SUPPLEMENTARY MATERIAL
