# Supplementary material for: What do ultrasound vocalizations really mean in rats with different origins of pain?
Source: Pain Rep. 2024 Dec 24;10(1):e1230. doi: 10.1097/PR9.0000000000001230 (PMC11671076; doi:10.1097/PR9.0000000000001230)
Supplement: SUPPLEMENTARY MATERIAL [file painreports-10-e1230-s001.pdf]

What do ultrasound vocalizations really mean in rats with different origins of pain?

Yang Yu[a]†, Chun-Li Li[a]†, Rui Du[a], Xiao-Liang Wang[a], Jun Chen[a,b]*

[a]Institute for Biomedical Sciences of Pain, Tangdu Hospital, The Fourth Military Medical University, Xi'an 710038, P. R. China
[b]Sanhang Institute for Brain Science and Technology, Northwestern Polytechnical University, Xi'an, 710129, P. R. China

Supplementary data

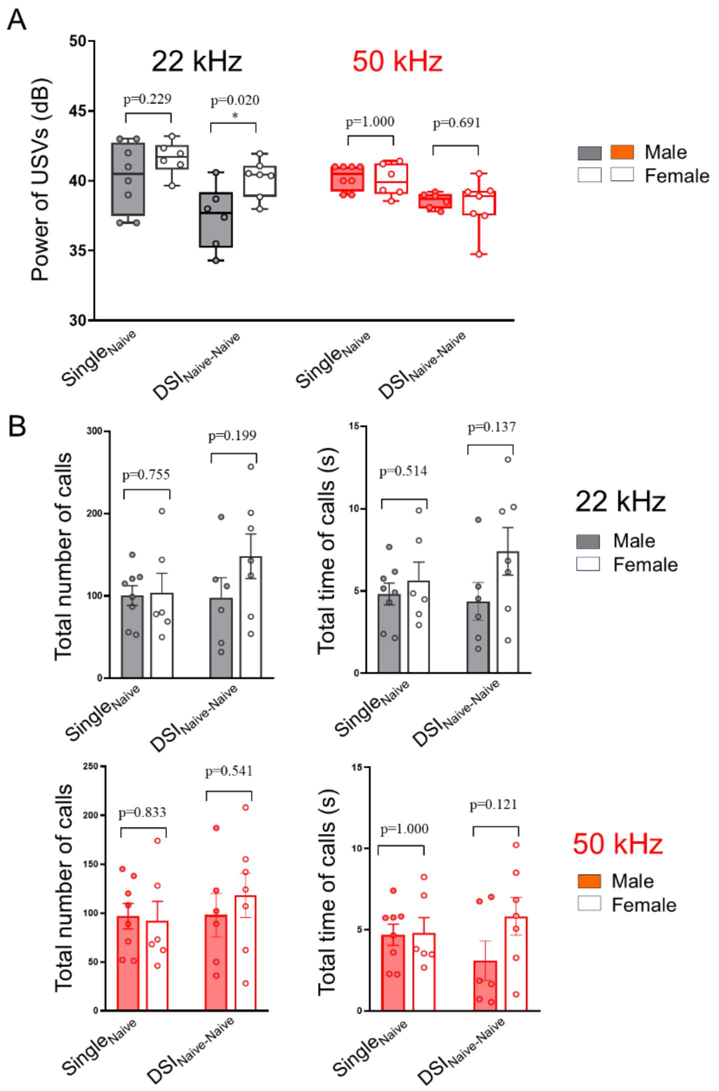

Fig.S1 Sex comparison of emissions of spontaneous USV calls. (A) comparison of powers of 22 kHz and 50 kHz spontaneous calls between male and female rats staying alone ($Single_{Naive}$) and those engaging dyadic social interaction (DSI) with a naïve conspecific ($DSI_{Naive\text{-}Naive}$). (B) comparison of the number and time of spontaneous calls between male and female rats under $Single_{Naive}$ and $DSI_{Naive\text{-}Naive}$ paradigms.

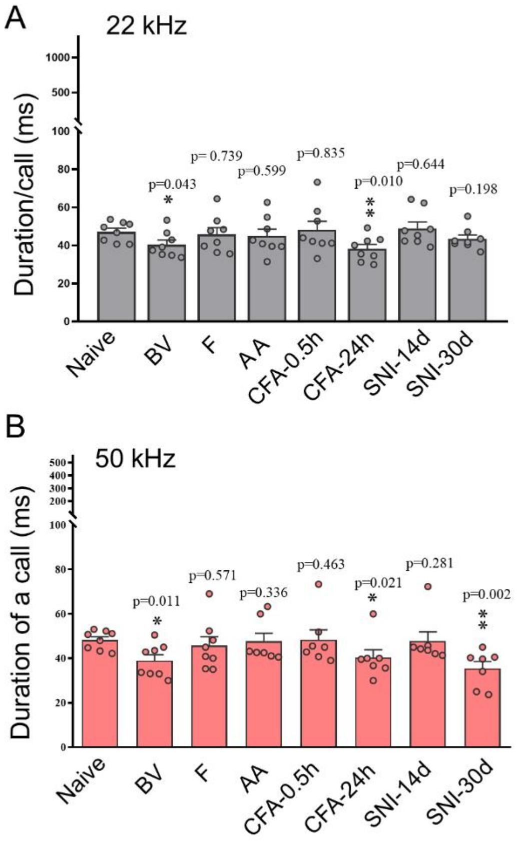

Fig.S2 Effects of various origins of pain on ultrasonographic signal duration in rats. (A) effects of pain on ultrasonographic signal duration of 22 kHz spontaneous calls. (B) effects of pain on ultrasonographic signal duration of 50 kHz spontaneous calls. Data expressed as Mean ± SEM; n=8 animals per group; *, p<0.05, **, p<0.01 vs. naïve or Veh.

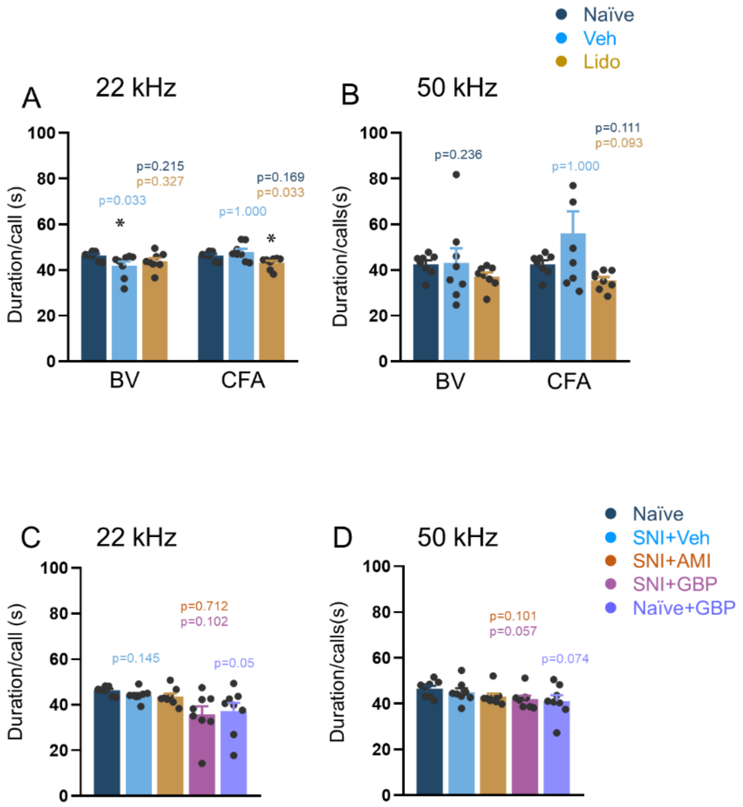

Fig.S3 Effects of antinociceptive, antidepressant and anticonvulsant on ultrasonographic signal duration in rats with peripheral inflammatory pain (A-B) or neuropathic pain (C-D). Lidocaine (Lido) was used to block the injection sites of BV and CFA, while amitriptyline (AMI) and gabapentin (GBP) were used to treat SNI-induced neuropathic pain. Data expressed as Mean ± SEM; n=8 animals per group; *, $p<0.05$ vs. Veh.

Table S1 Overview of the effects of different pain on audible and ultrasound vocalizations

Type	Type of pain	Type of Calls	rodents	References
Spontaneous calls	Orofacial formalin test	50 kHz (number) ↓	Male Wistar rats (250–300g)	Araya, et al., 2020
	Orofacial formalin test	22 kHz (number) ↑<br>50 kHz (number) -	Male SD rats (280–320 g)	Barroso, et al., 2019
	Paw formalin test	>30 kHz (number and duration) ↑	Male BALB/c mice (20–25 g)	Han, et al., 2013
	Forepaw formalin test	30±10 kHz (number) ↑	Male Wistar rats (210–320 g)	Oliveira and Barros, 2006
	CFA model	22 kHz (duration) -	Male SD rats (280–380 g)	Jourdan, et al., 2002
	Carrageenan model	22 kHz (duration) -	Male SD rats (280–380 g)	Jourdan, et al., 2002
	Arthritic pain	22 kHz (duration) -	Male SD rats (280–380 g)	Jourdan, et al., 2002
	CCI-ION	50 kHz (number) ↓	Male Wistar rats (280–380 g)	Araya, et al., 2022a; Araya, et al., 2022b
	CCI model	22 kHz ↑<br>50 kHz ↓	Male SD rats (250–350 g)	Burgdorf, et al., 2019 Ghoreishi-Haack, et al., 2018;
	SNI model	37 kHz (rate) ↑<br>50 kHz calls (rate) ↑	Male C57BL/6 and C3H/HeNCrl mice	Kurejova, et al., 2010
	SNI model	22 kHz (number) ↑	Male SD rats (180–210 g)	Kim, et al., 2014a; Lim, et al., 2014; Lim, et al., 2015; Lim, et al., 2016; Lim, et al., 2018; Lim, et al., 2019
	pSNL model	20-100 kHz (number) ↑	Male and female C57BL/6 mice	Abraham, et al., 2020
	LPS-induced cerebral inflammatory hyperalgesia	22 kHz (number and latency) ↓	Male SD rats (175–200 g)	Martino, et al., 2008

	Streptozotocin model	22 kHz (duration) -	Male SD rats (280–380 g)	Jourdan, et al.，2002
	Cancer pain	37 kHz (rate) ↑<br>50 kHz (rate) ↑	Male C57BL/6 and C3H/HeNCrl mice	Kurejova, et al.，2010
Evoked calls	Paw formalin test	22 kHz (duration) -	Male Lister–Hooded rats (240–340 g)	Olango, et al.，2012; Rea, et al.，2009
	Paw formalin test	22 kHz (number) -	Male Wistar rats (190–210 g)<br>Male C57BL/6 mice (20–25 g)	Wallace, et al.，2005
	Arthritic pain	22 kHz (duration) ↑	Male SD rats (220–270 g) (360–380 g)	Calvino, et al.，1996
	Arthritic pain	Audible vocalizations (20 Hz–16 kHz) (number and duration) ↑<br>USVs (25 ± 4 kHz) (number and duration) ↑<br>evoked by innocuous (500 g/30 mm^2) and noxious (2000 g/30 mm^2) mechanical stimuli	Male SD rats (230–280 g)<br>Male SD rats (140–170 g)	Adwanikar, et al.，2007; Cragg, et al.，2016;<br>Fu, et al.，2008a;<br>Fu, et al.，2008b;<br>Gregoire, et al.，2013;<br>Han, et al.，2005a;<br>Han, et al.，2005b;<br>Han, et al.，2005c;<br>Han, et al.，2010;<br>Medina, et al.，2014;<br>Mazzitelli, et al.，2019;<br>Palazzo, et al.，2008;<br>Shen, et al.，2022;<br>Tappe-Theodor, et al.，2011;<br>Thompson, et al.，2015
	Arthritic pain	50 kHz (number and total duration) ↑	Male SR-KO and WT mice	Tsuzuki, et al.，2012
	pSNL model	22 kHz (number) -	Male Wistar rats (190–210 g)	Wallace, et al.，2005
	SNL model	Audible	Male and	Mazzitelli, et al.，

	vocalizations (20 Hz–16 kHz) (total duration) ↑ USVs (25 ± 4 kHz) (total duration) ↑ evoked by innocuous or noxious stimuli	female SD rats (150–350 g)	2022; Presto, et al.， 2021; Thompson, et al.， 2018
Bladder inflammation model	22 kHz (number) -	Male Wistar rats (190–210 g)	Wallace, et al.， 2005

Notes: CCI, chronic constriction injury; CCI-ION, chronic constriction injury of the infraorbital nerve; CFA, Complete Freund's adjuvant; LPS, lipopolysaccharide; SD, Sprague-Dawley; SNI, spared nerve injury; SNL, spinal nerve ligation; pSNL, partial sciatic nerve ligation.

References:

[1] Abraham AD, Leung EJ, Wong BA, Rivera ZM, Kruse LC, Clark JJ, Land BB. Orally consumed cannabinoids provide long-lasting relief of allodynia in a mouse model of chronic neuropathic pain. Neuropsychopharmacol 2020;45:1105-14.

[2] Adwanikar H, Ji G, Li W, Doods H, Willis WD, Neugebauer V. Spinal CGRP1 receptors contribute to supraspinally organized pain behavior and pain-related sensitization of amygdala neurons. Pain 2007;132:53-66.

[3] Araya EI, Baggio DF, Koren LO, Andreatini R, Schwarting RKW, Zamponi GW, Chichorro JG. Acute orofacial pain leads to prolonged changes in behavioral and affective pain components. Pain 2020;161:2830-40.

[4] Araya EI, Baggio DF, Koren LO, Schwarting RKW, Chichorro JG. Trigeminal neuropathic pain reduces 50-kHz ultrasonic vocalizations in rats, which are restored by analgesic drugs. Eur J Pharmacol 2022;922:174905.

[5] Araya EI, Carvalho EC, Andreatini R, Zamponi GW, Chichorro JG. Trigeminal neuropathic pain causes changes in affective processing of pain in rats. Mol Pain 2022;18:1-11.

[6] Barroso AR, Araya EI, de Souza CP, Andreatini R, Chichorro JG. Characterization of rat ultrasonic vocalization in the orofacial formalin test: Influence of the social context. Eur Neuropsychopharm 2019;29:1213-26.

[7] Burgdorf JS, Ghoreishi-Haack N, Cearley CN, Kroes RA, Moskal JR. Rat ultrasonic vocalizations as a measure of the emotional component of chronic pain. Neuroreport 2019;30:863-66.

[8] Calvino B, Besson JM, Boehrer A, Depaulis A. Ultrasonic vocalization (22-28kHz) in a model of chronic pain, the arthritic rat: effects of analgesic drugs. Neuroreport 1996;7:581-84.

[9] Cragg B, Ji G, Neugebauer V. Differential contributions of vasopressin V1A and oxytocin receptors in the amygdala to pain-related behaviors in rats. Mol Pain 2016;12:1-07.

[10] Fu Y, Han J, Ishola T, Scerbo M, Adwanikar H, Ramsey C, Neugebauer V. PKA and ERK, but not PKC, in the amygdala contribute to pain-related synaptic plasticity and behavior. Mol Pain

2008;4:26-46.

[11] Fu Y, Neugebauer V. Differential mechanisms of CRF1 and CRF2 receptor functions in the amygdala in pain-related synaptic facilitation and behavior. J Neurosci 2008;28:3861-76.

[12] Ghoreishi-Haack N, Priebe JM, Aguado JD, Colechio EM, Burgdorf JS, Bowers MS, Cearley CN, Khan MA, Moskal JR. NYX-2925 is a novel N-methyl-d-aspartate receptor modulator that induces rapid and long-lasting analgesia in rat models of neuropathic pain. J Pharmacol Exp Ther 2018;366:485-97.

[13] Gregoire S, Neugebauer V. 5-HT_{2C}R blockade in the amygdala conveys analgesic efficacy to SSRIs in a rat model of arthritis pain. Mol Pain 2013;9:41-53.

[14] Han JS, Adwanikar H, Li Z, Ji G, Neugebauer V. Facilitation of synaptic transmission and pain responses by CGRP in the amygdala of normal rats. Mol Pain 2010;6:10-24.

[15] Han JS, Bird GC, Li W, Jones J, Neugebauer V. Computerized analysis of audible and ultrasonic vocalizations of rats as a standardized measure of pain-related behavior. J Neurosci Meth 2005;141:261-69.

[16] Han JS, Li W, Neugebauer V. Critical role of calcitonin gene-related peptide 1 receptors in the amygdala in synaptic plasticity and pain behavior. J Neurosci 2005;25:10717-28.

[17] Han JS, Neugebauer V. mGluR1 and mGluR5 antagonists in the amygdala inhibit different components of audible and ultrasonic vocalizations in a model of arthritic pain. Pain 2005;113:211-22.

[18] Han P, Zhao J, Liu SB, Yang CJ, Wang YQ, Wu GC, Xu DM, Mi WL. Interleukin-33 mediates formalin-induced inflammatory pain in mice. Neuroscience 2013;241:59-66.

[19] Jourdan D, Ardid D, Eschalier A. Analysis of ultrasonic vocalisation does not allow chronic pain to be evaluated in rats. Pain 2002;95:165-73.

[20] Kim JG, Lim DW, Cho S, Han D, Kim YT. The edible brown seaweed *Ecklonia cava* reduces hypersensitivity in postoperative and neuropathic pain models in rats. Molecules 2014;19:7669-78.

[21] Kurejova M, Nattenmuller U, Hildebrandt U, Selvaraj D, Stosser S, Kuner R. An improved behavioural assay demonstrates that ultrasound vocalizations constitute a reliable indicator of chronic cancer pain and neuropathic pain. Mol Pain 2010;6:18-25.

[22] Lim DW, Kim JG, Kim YT. Analgesic effect of indian gooseberry (*Emblica officinalis* fruit) extracts on postoperative and neuropathic pain in rats. Nutrients 2016;8:760-70.

[23] Lim DW, Kim JG, Han D, Kim YT. Analgesic effect of Harpagophytum procumbens on postoperative and neuropathic pain in rats. Molecules 2014;19:1060-68.

[24] Lim DW, Kim JG, Han T, Jung SK, Lim EY, Han D, Kim YT. Analgesic effect of *Ilex paraguariensis* extract on postoperative and neuropathic pain in rats. Biol Pharm Bull 2015;38:1573-79.

[25] Lim DW, Kim JG, Lim EY, Kim YT. Antihyperalgesic effects of ashwagandha (*Withania somnifera* root extract) in rat models of postoperative and neuropathic pain. Inflammopharmacology 2018;26:207-15.

[26] Lim EY, Kim JG, Lee J, Lee C, Shim J, Kim YT. Analgesic effects of *Cnidium officinale* extracts on postoperative, neuropathic, and menopausal pain in rat models. Evid-Based Compl Alt 2019;2019:1-08.

[27] Martino G, Perkins MN. Tactile-induced ultrasonic vocalization in the rat: a novel assay to assess anti-migraine therapies in vivo. Cephalalgia 2008;28:723-33.

[28] Mazzitelli M, Neugebauer V. Amygdala group II mGluRs mediate the inhibitory effects of systemic group II mGluR activation on behavior and spinal neurons in a rat model of arthritis pain. Neuropharmacology 2019;158:107706.
[29] Mazzitelli M, Yakhnitsa V, Neugebauer B, Neugebauer V. Optogenetic manipulations of CeA-CRF neurons modulate pain- and anxiety-like behaviors in neuropathic pain and control rats. Neuropharmacology 2022;210:109031.
[30] Medina G, Ji G, Gregoire S, Neugebauer V. Nasal application of neuropeptide S inhibits arthritis pain-related behaviors through an action in the amygdala. Mol Pain 2014;10:32-45.
[31] Olango WM, Roche M, Ford GK, Harhen B, Finn DP. The endocannabinoid system in the rat dorsolateral periaqueductal grey mediates fear-conditioned analgesia and controls fear expression in the presence of nociceptive tone. Brit J Pharmacol 2012;165:2549-60.
[32] Oliveira AR, Barros HMT. Ultrasonic rat vocalizations during the formalin test: a measure of the affective dimension of pain? Anesth Analg 2006;102:832-39.
[33] Palazzo E, Fu Y, Ji G, Maione S, Neugebauer V. Group III mGluR7 and mGluR8 in the amygdala differentially modulate nocifensive and affective pain behaviors. Neuropharmacology 2008;55:537-45.
[34] Presto P, Ji G, Junell R, Griffin Z, Neugebauer V. Fear extinction-based inter-individual and sex differences in pain-related vocalizations and anxiety-like behaviors but not nocifensive reflexes. Brain Sci 2021;11:1339-58.
[35] Rea K, Lang Y, Finn DP. Alterations in extracellular levels of gamma-aminobutyric acid in the rat basolateral amygdala and periaqueductal gray during conditioned fear, persistent pain and fear-conditioned analgesia. J Pain 2009;10:1088-98.
[36] Shen CL, Wang R, Yakhnitsa V, Santos JM, Watson C, Kiritoshi T, Ji G, Hamood AN, Neugebauer V. Gingerol-enriched ginger supplementation mitigates neuropathic pain via mitigating intestinal permeability and neuroinflammation: gut-brain connection. Front Pharmacol 2022;13:912609.
[37] Tappe-Theodor A, Fu Y, Kuner R, Neugebauer V. Homer1a signaling in the amygdala counteracts pain-related synaptic plasticity, mGluR1 function and pain behaviors. Mol Pain 2011;7:38-44.
[38] Thompson JM, Ji G, Neugebauer V. Small-conductance calcium-activated potassium (SK) channels in the amygdala mediate pain-inhibiting effects of clinically available riluzole in a rat model of arthritis pain. Mol Pain 2015;11:51-61.
[39] Thompson JM, Yakhnitsa V, Ji G, Neugebauer V. Small conductance calcium activated potassium (SK) channel dependent and independent effects of riluzole on neuropathic pain-related amygdala activity and behaviors in rats. Neuropharmacology 2018;138:219-31.
[40] Tsuzuki H, Maekawa M, Konno R, Hori Y. Functional roles of endogenous D-serine in pain-induced ultrasonic vocalization. Neuroreport 2012;23:937-41.
[41] Wallace VCJ, Norbury TA, Rice ASC. Ultrasound vocalisation by rodents does not correlate with behavioural measures of persistent pain. Eur J Pain 2005;9:445-52.

Table S2 Statistical analysis methods, normality and equal variance tests and *p* values for the relevant graphs

Figure number	group	n	Normality test	Equal variance test	Statistic method	*p* value	*t* value
Fig.2A Power of USVs	Single$_{\text{(Naive)}}$ *vs* DSI$_{\text{(Naïve-Naive)}}$ 22kHz	N=8 N=6	Passed	Passed	two-tailed *t*-test	*p* =0.064	*t* =2.22
					two-tailed M-W *U* test		
	DSI$_{\text{(Naïve-Naive)}}$ *vs* DSI$_{\text{(Naïve-Pain)}}$ 22kHz	N=6	Passed	Passed	two-tailed *t*-test	*p* =0.179	*t* =1.445
					two-tailed M-W *U* test		
	Single$_{\text{(Naive)}}$ *vs* DSI$_{\text{(Naïve-Naive)}}$ 50kHz	N=8 N=6	Failed	-	two-tailed *t*-test		
					two-tailed M-W *U* test	*p* =0.005	*U*=3
	DSI$_{\text{(Naïve-Naive)}}$ *vs* DSI$_{\text{(Naïve-Pain)}}$ 50kHz	N=6	Passed	Passed	two-tailed *t*-test	*p* =0.479	*t* =0.752
					two-tailed M-W *U* test		
Fig.2B Power of USVs	BV *vs* Naive	N=8	Failed	-	two-tailed *t*-test		
					two-tailed M-W *U* test	*p* =0.065	*U* =14
	Formalin *vs* Naive	N=8	Failed	-	two-tailed *t*-test		
					two-tailed M-W *U* test	*p* =0.645	*U*=27
	Acetic acid *vs* Naive	N=8	Passed	Passed	two-tailed *t*-test	*p* =0.563	*t* =0.592
					two-tailed M-W *U* test		
	CFA-30min *vs* Naive	N=8	Passed	Passed	two-tailed *t*-test	*p* =0.242	*t* <0.001
					two-tailed M-W *U* test		
	CFA-24h *vs* Naive	N=8	Passed	Passed	two-tailed *t*-test	*p* =0.431	*t* =0.812
					two-tailed M-W *U* test		
	SNI-14d *vs* Naive	N=8	Passed	Failed	two-tailed *t*-test		

					two-tailed M-W *U* test	*p* =0.574	*U*=37.5
	SNI-30d *vs* Naive	N=8	Passed	Passed	two-tailed *t*-test	*p* =1.0	*t* <0.001
					two-tailed M-W *U* test		
Fig.2C Power of USVs	BV *vs* Naive	N=8	Failed	-	two-tailed *t*-test		
					two-tailed M-W *U* test	*p* =0.007	*U* =7
	Formalin *vs* Naive	N=8	Failed	-	two-tailed *t*-test		
					two-tailed M-W *U* test	*p* =0.005	*U*=6
	Acetic acid *vs* Naive	N=8	Failed	-	two-tailed *t*-test		
					two-tailed M-W *U* test	*p* =0.328	*U*=22
	CFA-30min *vs* Naive	N=8	Failed	-	two-tailed *t*-test		
					two-tailed M-W *U* test	*p* =0.159	*U*=19
	CFA-24h *vs* Naive	N=8	Failed	-	two-tailed *t*-test		
					two-tailed M-W *U* test	*p* =0.002	*U*=4
	SNI-14d *vs* Naive	N=8	Failed	-	two-tailed *t*-test		
					two-tailed M-W *U* test	*p* =0.798	*U*=29
	SNI-30d *vs* Naive	N=8	Failed	-	two-tailed *t*-test		
					two-tailed M-W *U* test	*p* =0.021	*U*=10
Fig.3A Total number of calls (22 kHz)	Single$_{(Naive)}$ *vs* DSI$_{(Naïve-Naive)}$	N=8 N=6	Passed	Passed	two-tailed *t*-test	*p* =0.912	*t* =0.113
					two-tailed M-W *U* test		
	DSI$_{(Naïve-Naive)}$ *vs* DSI$_{(Naïve-Pain)}$	N=6	Passed	Passed	two-tailed *t*-test	*p* =0.217	*t* =1.317
					two-tailed M-W *U* test		
Fig.3A	Single$_{(Naive)}$	N=8	Passed	Passed	two-tailed *t*-	*p*	*t*

Total time of calls (22 kHz)	*vs* DSI$_{\text{(Naïve-Naive)}}$	N=6			test	=0.726	=0.113
					two-tailed M-W *U* test		
	DSI$_{\text{(Naïve-Naive)}}$ *vs* DSI$_{\text{(Naïve-Pain)}}$	N=6	Failed	-	two-tailed *t*-test		
					two-tailed M-W *U* test	*p* =0.394	*U*=12
Fig.3B Total number of calls (50 kHz)	Single$_{\text{(Naive)}}$ *vs* DSI$_{\text{(Naïve-Naive)}}$	N=8 N=6	Passed	Passed	two-tailed *t*-test	*p* =0.965	*t* =0.045
					two-tailed M-W *U* test		
	DSI$_{\text{(Naïve-Naive)}}$ *vs* DSI$_{\text{(Naïve-Pain)}}$	N=6	Passed	Passed	two-tailed *t*-test	*p* =0.599	*t* =0.543
					two-tailed M-W *U* test		
Fig.3B Total time of calls (50 kHz)	Single$_{\text{(Naive)}}$ *vs* DSI$_{\text{(Naïve-Naive)}}$	N=8 N=6	Failed	-	two-tailed *t*-test		
					two-tailed M-W *U* test	*p* =0.228	*U*=14
	DSI$_{\text{(Naïve-Naive)}}$ *vs* DSI$_{\text{(Naïve-Pain)}}$	N=6	Failed	-	two-tailed *t*-test		
					two-tailed M-W *U* test	*p* =0.394	*U*=24
Fig.4A Total number of calls (22 kHz)	BV *vs* Naive	N=8	Passed	Passed	two-tailed *t*-test	*p* =0.001	*t* =3.95
					two-tailed M-W *U* test		
	Formalin *vs* Naive	N=8	Passed	Passed	two-tailed *t*-test	*p* <0.001	*t* =4.621
					two-tailed M-W *U* test		
	Acetic acid *vs* Naive	N=8	Passed	Passed	two-tailed *t*-test	*p* <0.001	*t* =5.782
					two-tailed M-W *U* test		
	CFA-30min *vs* Naive	N=8	Passed	Passed	two-tailed *t*-test	*p* <0.001	*t* =6.423
					two-tailed M-W *U* test		
	CFA-24h *vs* Naive	N=8	Failed	-	two-tailed *t*-test		
					two-tailed M-W *U* test	*p* =0.003	*U* =5.5

	SNI-14d *vs* Naive	N=8	Passed	Passed	two-tailed *t*-test	*p* =0.015	*t* =2.767
					two-tailed M-W *U* test		
	SNI-30d *vs* Naive	N=8	Passed	Passed	two-tailed *t*-test	*p* <0.001	*t* =4.988
					two-tailed M-W *U* test		
Fig.4B Total number of calls (50 kHz)	BV *vs* Naive	N=8	Passed	Passed	two-tailed *t*-test	*p* =0.002	*t* = 3.791
					two-tailed M-W *U* test		
	Formalin *vs* Naive	N=8	Passed	Failed	two-tailed *t*-test		
					two-tailed M-W *U* test	*p* <0.001	*U*=1.5
	Acetic acid *vs* Naive	N=8	Passed	Failed	two-tailed *t*-test		
					two-tailed M-W *U* test	*p* <0.001	*U*=0
	CFA-30min *vs* Naive	N=8	Passed	Failed	two-tailed *t*-test		
					two-tailed M-W *U* test	*p* <0.001	*U*=0
	CFA-24h *vs* Naive	N=8	Failed	-	two-tailed *t*-test		
					two-tailed M-W *U* test	*p* <0.001	*U*=0
	SNI-14d *vs* Naive	N=8	Passed	Passed	two-tailed *t*-test	*p*=0.037	*t* =2.318
					two-tailed M-W *U* test		
	SNI-30d *vs* Naive	N=8	Passed	Failed	two-tailed *t*-test		
					two-tailed M-W *U* test	*p* <0.001	*U*=0
Fig.4C Total time of calls (s) (22 kHz)	BV *vs* Naive	N=8	Passed	Passed	two-tailed *t*-test	*p* =0.002	*t* = 3.780
					two-tailed M-W *U* test		
	Formalin *vs* Naive	N=8	Passed	Passed	two-tailed *t*-test	*p* =0.001	*t* = 4.254
					two-tailed		

					M-W *U* test		
	Acetic acid *vs* Naive	N=8	Passed	Passed	two-tailed *t*-test	$p<0.001$	$t = 4.816$
					two-tailed M-W *U* test		
	CFA-30min *vs* Naive	N=8	Passed	Failed	two-tailed *t*-test		
					two-tailed M-W *U* test	$p<0.001$	U=0
	CFA-24h *vs* Naive	N=8	Failed	-	two-tailed *t*-test		
					two-tailed M-W *U* test	$p=0.003$	U=5
	SNI-14d *vs* Naive	N=8	Passed	Passed	two-tailed *t*-test	p=0.015	t =2.772
					two-tailed M-W *U* test		
	SNI-30d *vs* Naive	N=8	Passed	Passed	two-tailed *t*-test	$p<0.001$	t=4.561
					two-tailed M-W *U* test		
Fig.4D Total time of calls (s) (50 kHz)	BV *vs* Naive	N=8	Passed	Passed	two-tailed *t*-test	p=0.002	t=3.929
					two-tailed M-W *U* test		
	Formalin *vs* Naive	N=8	Passed	Failed	two-tailed *t*-test		
					two-tailed M-W *U* test	p=0.001	U=2
	Acetic acid *vs* Naive	N=8	Passed	Failed	two-tailed *t*-test		
					two-tailed M-W *U* test	$p<0.001$	U=0
	CFA-30min *vs* Naive	N=8	Passed	Failed	two-tailed *t*-test		
					two-tailed M-W *U* test	$p<0.001$	U=0
	CFA-24h *vs* Naive	N=8	Failed	-	two-tailed *t*-test		
					two-tailed M-W *U* test	$p<0.001$	U=0
	SNI-14d *vs* Naive	N=8	Passed	Passed	two-tailed *t*-test	p=0.019	t =2.668

					two-tailed M-W *U* test		
	SNI-30d *vs* Naive	N=8	Passed	Failed	two-tailed *t*-test		
					two-tailed M-W *U* test	*p* =0.001	*U*=1.5

Figure number	group	n	Statistic method	Post hoc multiple comparisons test
Fig.5A Call rate 22kHz calls	Veh + BV *vs* lidocaine +BV	N=8	Repeated-measures (F=1.045; *p*=0.398)	t5: *p*=0.016
				t10: *p*=0.145
				t15: *p*=0.701
				t20: *p*=0.179
				t25: *p*=0.291
				t30: *p*=0.035
Fig.5B Call rate 50kHz calls	Veh + BV *vs* lidocaine +BV	N=8	Repeated-measures (F=1.045; *p*=0.398)	t5: *p*=0.077
				t10: *p*=0.376
				t15: *p*=0.599
				t20: *p*=0.470
				t25: *p*=0.306
				t30: *p*=0.033
Fig.5C Call rate 22kHz calls	Veh + CFA *vs* lidocaine +CFA	N=8	Repeated-measures (F=10.454; *p*<0.001)	t5: *p*=0.172
				t10: *p*=0.039
				t15: *p*=0.314
				t20: *p*=0.01
				t25: *p*=0.202
				t30: *p*=0.687
Fig.5D Call rate 50kHz calls	Veh + CFA *vs* lidocaine +CFA	N=8	Repeated-measures (F=12.340; *p*<0.001)	t5: *p*=0.974
				t10: *p*=0.121
				t15: *p*=0.137
				t20: *p*=0.043
				t25: *p*=0.218
				t30: *p*=1.0
Fig.5E Call rate 22kHz calls	SNI+ Veh *vs* SNI+ AMI	N=8	Repeated-measures (F=21.553; *p*<0.001)	t5: *p*=0.037
				t10: *p*=0.003
				t15: *p*=0.014
				t20: *p*=0.031
				t25: *p*=0.407
				t30: *p*=0.812
Fig.5E Call rate 22kHz calls	SNI+ Veh *vs* SNI+ GBP	N=8	Repeated-measures	t5: *p*=0.045
				t10: *p*=0.384

			(F=21.553; *p*<0.001)	t15: *p*=0.328
				t20: *p*=0.060
				t25: *p*=0.067
				t30: *p*=0.001
Fig.5F Call rate 50kHz calls	SNI+ Veh *vs* SNI+ AMI	N=8	Repeated-measures (F=20.932; *p*<0.001)	t5: *p*=0.039
				t10: *p*=0.003
				t15: *p*=0.007
				t20: *p*=0.046
				t25: *p*=0.292
				t30: *p*=0.467
Fig.5F Call rate 50kHz calls	SNI+ Veh *vs* SNI+ GBP	N=8	Repeated-measures (F=20.932; *p*<0.001)	t5: *p*=0.159
				t10: *p*=0.386
				t15: *p*=0.094
				t20: *p*=0.126
				t25: *p*=0.012

Figure number	group	n	Normality test	Equal variance test	Statistic method	Post hoc multiple comparisons test
Fig.6D Total number of calls (analgesia) 22kHz calls	Naïve *vs* Veh +BV *vs* lidocaine +BV	N=8	Failed	-	Kruskal-Wallis (*K*=13.671; *p*=0.001)	saline+BV *vs* lidocaine+BV *p*=0.018 saline+BV *vs* Naive *p*=0.001 lidocaine+BV *vs* naïve *p*=1.0
	Naïve *vs* Veh +CFA *vs* lidocaine +CFA	N=8	Failed	-	Kruskal-Wallis (*K*=10.999; *p*=0.004)	saline+CFA *vs* lidocaine+CFA *p*=0.04 saline+CFA *vs* Naive *p*=0.005 lidocaine+CFA *vs* naïve *p*=1.0
Fig.5F Total number of calls (analgesia) 50kHz calls	Naïve *vs* Veh +BV *vs* lidocaine +BV	N=8	passed	Passed	One-way ANOVA (F=5.676 ;*p*=0.011)	saline+BV *vs* lidocaine+BV *p*=0.045 saline+BV *vs* Naive *p*=0.03 lidocaine+BV *vs* naïve *p*=1.0

	Naïve *vs* Veh +CFA *vs* lidocaine +CFA	N=8	passed	Passed	One-way ANOVA (F=4.593 ;*p*=0.022)	saline+CFA *vs* lidocaine+CFA *p*=0.157 saline+CFA *vs* Naive *p*=0.006 lidocaine+CFA *vs* naïve *p*=0.133
Fig.6E Total time of calls(s) (analgesia) 22kHz calls	Naïve *vs* Veh +BV *vs* lidocaine +BV	N=8	Failed	-	Kruskal-Wallis (*K*=13.484;*p*=0.001	saline+BV *vs* lidocaine+BV *p*=0.02 saline+BV *vs* Naive *p*=0.001 lidocaine+BV *vs* naïve *p*=1.0
	Naïve *vs* Veh +CFA *vs* lidocaine +CFA	N=8	Failed	-	Kruskal-Wallis (*K*=10.518 ;*p*=0.005	saline+CFA *vs* lidocaine+CFA *p*=0.085 saline+CFA *vs* Naive *p*=0.005 lidocaine+CFA *vs* naïve *p*=0.992
Fig.6G Total time of calls (s) (analgesia) 50kHz calls	Naïve *vs* Veh +BV *vs* lidocaine +BV	N=8	passed	passed	One-way ANOVA (F=5.766 ;*p*=0.01)	saline+BV *vs* lidocaine+BV *p*=0.061 saline+BV *vs* Naive *p*=0.003 lidocaine+BV *vs* naïve *p*=0.175
	Naïve *vs* Veh +CFA *vs* lidocaine +CFA	N=8	Passed	Passed	One-way ANOVA (F=3.755;*p*=0.04)	saline+CFA *vs* lidocaine+CFA *p*=0.334 saline+CFA *vs* Naive *p*=0.013 lidocaine+CFA *vs* naïve *p*=0.1
Fig.7C Total number of calls (analgesia) 22kHz calls	Naïve *vs* SNI +Veh *vs* SNI +AMI	N=8	Passed	Passed	One-way ANOVA (F=10.141; *p*=0.001)	Naïve *vs* SNI+Veh *p*=0.028 SNI+Veh *vs* SNI+AMI *p*<0.001 Naïve *vs*

						SNI+AMI<br>*p*=0.044	
	Naïve *vs* SNI +Veh *vs vs* SNI +GBP	N=8	Failed	-	Kruskal-Wallis (*K*=13.335; *p*=0.001	Naïve *vs* SNI+Veh<br>*p*=0.013<br>SNI+Veh *vs* SNI+GBP<br>*p*=0.002<br>Naïve *vs* SNI+GBP<br>*p*=1.0	
	Naïve *vs* Naïve +GBP	N=8	Passed	Passed	two-tailed *t*-test	*p* =0.320	*t* =1.032
					two-tailed M-W *U* test		
Fig.7E Total number of calls (analgesia) 50kHz calls	Naïve *vs* SNI +Veh *vs* SNI +AMI	N=8	Passed	Passed	One-way ANOVA (F=7.938; *p*=0.003)	Naïve *vs* SNI+Veh<br>*p*=0.038<br>SNI+Veh *vs* SNI+AMI<br>*p*=0.001<br>Naïve *vs* SNI+AMI<br>*p*=0.094	
	Naïve *vs* SNI +Veh *vs vs* SNI +GBP	N=8	Passed	Passed	One-way ANOVA (F=4.265; *p*=0.028)	Naïve *vs* SNI+Veh<br>*p*=0.017<br>SNI+Veh *vs* SNI+GBP<br>*p*=0.023<br>Naïve *vs* SNI+GBP<br>*p*=0.883	
	Naïve *vs* Naïve +GBP	N=8	Passed	Passed	two-tailed *t*-test	*p* =0.816	*t* =0.238
					two-tailed M-W *U* test		

Fig.7D<br>Total time of calls (s)<br>(analgesia)<br>22kHz calls	Naïve *vs*<br>SNI +Veh<br>*vs* SNI<br>+AMI	N=8	Failed	-	Kruskal-Wallis<br>(*K*=13.145; *p*=0.001	Naïve *vs*<br>SNI+Veh<br>*p*=0.014<br>SNI+Veh *vs*<br>SNI+AMI<br>*p*=0.002<br>Naïve *vs*<br>SNI+AMI<br>*p*=1.0	
	Naïve *vs*<br>SNI +Veh<br>*vs vs* SNI<br>+GBP	N=8	Failed	-	Kruskal-Wallis<br>(*K*=13.320; *p*=0.001	Naïve *vs*<br>SNI+Veh<br>*p*=0.007<br>SNI+Veh *vs*<br>SNI+GBP<br>*p*=0.003<br>Naïve *vs*<br>SNI+GBP<br>*p*=1.0	
	Naïve *vs*<br>Naïve<br>+GBP	N=8	Failed	-	two-tailed *t*-test		
					two-tailed M-W *U* test	*p* =0.382	*U*=41
Fig.7FTotal time of calls (s)<br>(analgesia)<br>50kHz calls	Naïve *vs*<br>SNI +Veh<br>*vs* SNI<br>+AMI	N=8	Passed	Passed	One-way ANOVA<br>(F=6.898; *p*=0.005)	Naïve *vs*<br>SNI+Veh<br>*p*=0.025<br>SNI+Veh *vs*<br>SNI+AMI<br>*p*=0.001<br>Naïve *vs*<br>SNI+AMI<br>*p*=0.231	
	Naïve *vs*<br>SNI +Veh<br>*vs vs* SNI<br>+GBP	N=8	Passed	Passed	One-way ANOVA<br>(F=3.561; *p*=0.047)	Naïve *vs*<br>SNI+Veh<br>*p*=0.023<br>SNI+Veh *vs*<br>SNI+GBP<br>*p*=0.045<br>Naïve *vs*<br>SNI+GBP<br>*p*=0.743	
	Naïve *vs*<br>Naïve<br>+GBP	N=8	Passed	Passed	two-tailed *t*-test	*p* =0.868	*t* =0.169
					two-tailed M-W *U* test		

Figure number	group	n	Normality test	Equal variance test	Statistic method	*p* value	*t* value
Fig.S1A Power of USVs	Male *vs* Female Single (Naive)22kHz	N=8 N=6	Passed	Passed	two-tailed *t*-test	*p* =0.229	*t* =1.267
					two-tailed M-W *U* test		
	Male *vs* Female DSI(Naïve-Naive) 22kHz	N=6 N=7	Passed	Passed	two-tailed *t*-test	*p* =0.02	*t* =2.727
					two-tailed M-W *U* test		
	Male *vs* Female Single (Naive)50kHz	N=8 N=6	Failed	-	two-tailed *t*-test		
					two-tailed M-W *U* test	*p* =1.0	*U*=24
	Male *vs* Female DSI(Naïve-Naive)50kHz	N=6	Passed	Passed	two-tailed *t*-test	*p* =0.691	*t* =0.480
					two-tailed M-W *U* test		
Fig.S1B Total number of calls 22kHz	Male *vs* Female Single (Naive)	N=8 N=6	Failed	-	two-tailed *t*-test		
					two-tailed M-W *U* test	*p* =0.755	*U*=21
	Male *vs* Female DSI(Naïve-Naive)	N=6 N=7	Passed	Passed	two-tailed *t*-test	*p* =0.199	*t* =1.367
					two-tailed M-W *U* test		
Fig.S1B Total time of calls 22kHz	Male *vs* Female Single (Naive)	N=8 N=6	Passed	Passed	two-tailed *t*-test	*p* =0.514	*t* =0.672
					two-tailed M-W *U* test		
	Male *vs* Female DSI(Naïve-Naive)	N=6 N=7	Passed	Passed	two-tailed *t*-test	*p* =0.137	*t* =1.605
					two-tailed M-W *U* test		
Fig.S1B Total number of calls 50kHz	Male *vs* Female Single (Naive)	N=8 N=6	Passed	Passed	two-tailed *t*-test	*p* =0.833	*t* =0.215
					two-tailed M-W *U* test		
	Male *vs* Female DSI(Naïve-Naive)	N=6 N=7	Passed	Passed	two-tailed *t*-test	*p* =0.541	*t* =0.632
					two-tailed M-W *U* test		
Fig.S1B	Male *vs*	N=8	Failed	-	two-tailed *t*-test		

Total time of calls 50kHz	Female Single (Naive)	N=6			two-tailed M-W *U* test	*p* =1.0	*U*=24
	Male *vs* Female DSI(Naïve-Naive)	N=6 N=7	Failed	-	two-tailed *t*-test		
					two-tailed M-W *U* test	*p* =0.121	*U*=42
Fig.S2A Duration /call (ms) 22kHz	BV *vs* Naive	N=8	Passed	Passed	two-tailed *t*-test	p=0.043	t=2.228
					two-tailed M-W *U* test		
	Formalin *vs* Naive	N=8	Passed	Passed	two-tailed *t*-test	p=0.739	t=0.340
					two-tailed M-W *U* test		
	Acetic acid *vs* Naive	N=8	Passed	Passed	two-tailed *t*-test	p=0.599	t=0.537
					two-tailed M-W *U* test		
	CFA-30min *vs* Naive	N=8	Passed	Passed	two-tailed *t*-test	p=0.835	t=0.212
					two-tailed M-W *U* test		
	CFA-24h *vs* Naive	N=8	Passed	Passed	two-tailed *t*-test	p=0.01	t=2.997
					two-tailed M-W *U* test		
	SNI-14d *vs* Naive	N=8	Passed	Passed	two-tailed *t*-test	p=0.644	t=0.211
					two-tailed M-W *U* test		
	SNI-30d *vs* Naive	N=8	Passed	Passed	two-tailed *t*-test	p=0.198	t=0.713
					two-tailed M-W *U* test		
Fig.S2B Duration of a call (ms) 50kHz	BV *vs* Naive	N=8	Passed	Failed	two-tailed *t*-test	*p* =0.011	*t* =3.047
					two-tailed M-W *U* test		
	Formalin *vs* Naive	N=8	Passed	Passed	two-tailed *t*-test	p=0.571	t=0.580
					two-tailed M-W *U* test		
	Acetic acid *vs* Naive	N=8	Failed	-	two-tailed *t*-test		
					two-tailed M-W *U* test	*p* =0.336	U=19
	CFA-30min *vs* Naive	N=8	Failed	-	two-tailed *t*-test		
					two-tailed M-W *U* test	*p*=0.463	U=21
	CFA-24h *vs* Naive	N=8	Failed	-	two-tailed *t*-test		
					two-tailed M-W *U*	*p*	*U*=8

					test	=0.021	
	SNI-14d *vs* Naive	N=8	Failed	-	two-tailed *t*-test		
					two-tailed M-W *U* test	*p*=0.281	U=18
	SNI-30d *vs* Naive	N=8	Passed	Passed	two-tailed *t*-test	*p* =0.002	t=3.824
					two-tailed M-W *U* test		

Fig.S3A Duration /call (s) (analgesia) 22kHz calls	Naïve *vs* Veh +BV *vs* lidocaine +BV	N=8	Passed	Passed	One-way ANOVA (F=2.616;*p*=0.097)	saline+BV *vs* lidocaine+BV *p*=0.327 saline+BV *vs* Naive *p*=0.033 lidocaine+BV *vs* naïve *p*=0.215
	Naïve *vs* Veh +CFA *vs* lidocaine +CFA	N=8	Failed	-	Kruskal-Wallis (*K*=7.023; *p*=0.03	saline+CFA *vs* lidocaine+CFA *p*=0.033 saline+CFA *vs* Naive *p*=1.0 lidocaine+CFA *vs* naïve *p*=0.169
Fig.S3B Duration /call (s) (analgesia) 50kHz calls	Naïve *vs* Veh +BV *vs* lidocaine +BV	N=8	passed	Failed	Kruskal-Wallis (*K*=2.885; *p*=0.236	-
	Naïve *vs* Veh +CFA *vs* lidocaine +CFA	N=8	passed	Failed	Kruskal-Wallis (*K*=6.005; *p*=0.05	saline+CFA *vs* lidocaine+CFA *p*=0.093 saline+CFA *vs* Naive *p*=1.0 lidocaine+CFA *vs* naïve *p*=0.111
Fig.S3C Duration /calls (s) (analgesia) 22kHz calls	Naïve *vs* SNI +Veh *vs* SNI +AMI	N=8	Passed	Passed	One-way ANOVA (F=2.002; *p*=0.160)	Naïve *vs* SNI+Veh *p*=0.145 SNI+Veh *vs* SNI+AMI *p*=0.712

						Naïve *vs* SNI+AMI *p*=0.073	
	Naïve *vs* SNI +Veh *vs* *vs* SNI +GBP	N=8	Passed	Failed	Kruskal-Wallis (*K*=10.500; *p*=0.005)	Naïve *vs* SNI+Veh *p*=0.867 SNI+Veh *vs* SNI+GBP *p*=0.102 Naïve *vs* SNI+GBP *p*=0.004	
	Naïve *vs* Naïve +GBP	N=8	Passed	Failed	two-tailed *t*-test	*p* =0.040	*t* =2.483
					two-tailed M-W *U* test		
Fig.S3D Duration /call (s) (analgesia) 50kHz calls	Naïve *vs* SNI +Veh *vs* SNI +AMI	N=8	Failed	-	Kruskal-Wallis (*K*=4.580; *p*=0.101	-	
	Naïve *vs* SNI +Veh *vs* *vs* SNI +GBP	N=8	Failed	-	Kruskal-Wallis (*K*=5.735; *p*=0.057	-	
	Naïve *vs* Naïve +GBP	N=8	Passed	Passed	two-tailed *t*-test	*p* =0.074	*t*=1.927
					two-tailed M-W *U* test		
